# Influence of HAART on Alternative Reading Frame Immune Responses over the Course of HIV-1 Infection

**DOI:** 10.1371/journal.pone.0039311

**Published:** 2012-06-29

**Authors:** Stephane Champiat, Rui André Saraiva Raposo, Nicholas J. Maness, John L. Lehman, Sean E. Purtell, Aaron M. Hasenkrug, Jacob C. Miller, Hansi Dean, Wayne C. Koff, Marisa Ailin Hong, Jeffrey N. Martin, Steven G. Deeks, Gerald E. Spotts, Christopher D. Pilcher, Fredrick M. Hecht, Esper G. Kallas, Keith E. Garrison, Douglas F. Nixon

**Affiliations:** 1 Division of Experimental Medicine, Department of Medicine, University of California San Francisco, San Francisco, California, United States of America; 2 Department of Pathology and Laboratory Medicine, University of Wisconsin-Madison, Madison, Wisconsin, United States of America; 3 International AIDS Vaccine Initiative, New York, New York, United States of America; 4 Division of Clinical Immunology and Allergy, University of São Paulo, São Paulo, Brazil, and Institute Adolfo Lutz, São Paulo, Brazil; 5 University of São Paulo, São Paulo, Brazil, Division of Clinical Immunology and Allergy, University of São Paulo, São Paulo, Brazil; 6 Epidemiology and Prevention Interventions Center, Division of Infectious Diseases, and The Positive Health Program, San Francisco General Hospital, University of California San Francisco, San Francisco, California, United States of America; 7 Positive Health Program, Department of Medicine, San Francisco General Hospital, University of California San Francisco, San Francisco, California, United States of America; 8 Department of Biology, Saint Mary’s College of California, Moraga, California, United States of America; University of Alabama, United States of America

## Abstract

**Background:**

Translational errors can result in bypassing of the main viral protein reading frames and the production of alternate reading frame (ARF) or cryptic peptides. Within HIV, there are many such ARFs in both sense and the antisense directions of transcription. These ARFs have the potential to generate immunogenic peptides called cryptic epitopes (CE). Both antiretroviral drug therapy and the immune system exert a mutational pressure on HIV-1. Immune pressure exerted by ARF CD8^+^ T cells on the virus has already been observed *in vitro*. HAART has also been described to select HIV-1 variants for drug escape mutations. Since the mutational pressure exerted on one location of the HIV-1 genome can potentially affect the 3 reading frames, we hypothesized that ARF responses would be affected by this drug pressure *in vivo*.

**Methodology/Principal findings:**

In this study we identified new ARFs derived from sense and antisense transcription of HIV-1. Many of these ARFs are detectable in circulating viral proteins. They are predominantly found in the HIV-1 env nucleotide region. We measured T cell responses to 199 HIV-1 CE encoded within 13 sense and 34 antisense HIV-1 ARFs. We were able to observe that these ARF responses are more frequent and of greater magnitude in chronically infected individuals compared to acutely infected patients, and in patients on HAART, the breadth of ARF responses increased.

**Conclusions/Significance:**

These results have implications for vaccine design and unveil the existence of potential new epitopes that could be included as vaccine targets.

## Introduction

CD8^+^ T cell responses are a major component in the immune control of HIV-1 replication [1], [2], [3], [4]. Primate studies suggest that the breadth and magnitude of the vaccine-induced CD8^+^ T cell response correlates with viral load [5]. In the Step Trial that failed to demonstrate efficacy, the Merck trivalent vaccine induced a low number of CD8^+^ T cell epitope responses per vaccinee (with a median of three epitopes), suggesting that to be effective, vaccines should induce a greater number of T cell responses [6].

HIV-1 infected cells are recognized by the CD8^+^ T cell receptor (TCR) through viral peptides presented on MHC-I molecules. Most of the HIV-1 epitopes described have been identified in HIV-1 proteins encoded by primary open reading frames (ORFs) of the viral genome [7], [8], [9], [10], [11], [12]. Aside from these “traditional epitopes”, a new type of epitopes derived from frame-shifted proteins, called alternative reading frame (ARF) epitopes or cryptic epitopes (CE), have been reported for influenza virus [13], [14], malignancies [15], [16], [17], [18], and an autoimmune disease [19]. ARF expression may be explained by several transcriptional and translational mechanisms [20]: ribosomal frame-shifting [21], cryptic promoter activation, internal ribosomal entry sites [22], initiation codon scan-through [23], doublet decoding alternative splicing patterns [24], and initiation from non-AUG codons [25].

These nontraditional Cytotoxic T Lymphocyte (CTL) epitopes are generated during HIV-1 and/or SIV infections [26], [27], [28], [29], [30], [31], [32]. However, another source of cryptic epitopes originates from antisense (3′ to 5′) RNA transcription - such antisense transcripts have been identified in HIV-1 infections [33], [34], [35], and CEs derived from these antisense RNA have been previously reported in SIV and HIV-1 infections [26], [28], [30]. CD8^+^ specific T cells against HIV-1 CE could potentially contribute to viral control *in vivo* [36]. *In vitro* studies show that SIV ARF epitope–specific CTLs are able to select for viral escape variants [30]. These ARF-derived peptides could therefore be an important source of epitopes and could be used in an HIV-1 vaccine to broaden the spectrum of the CD8^+^ T cell responses.

To date, many studies have shown that immunological pressure exerted by CTL shapes HIV-1 sequences by selecting for CTL escape mutations [32], [37], [38], [39]. HAART has also been described to select HIV-1 variants for drug escape mutations [40], [41], [42], [43], [44], [45], [46], [47], [48]. More recently, there has been some evidence that emergence of drug resistance mutations can potentially abolish HIV-1 CTL responses [49].

In this regard, we anticipated that ARFs would be affected by immunological and drug pressure *in vivo*. Immune pressure exerted by the ARF CD8^+^ T cells on the virus has already been reported *in* *vitro* [30]. Since the mutational pressure exerted on one location of HIV-1’s genome can potentially affect each of the 3 reading frames, ARF expression could be affected by HAART. HAART mutational pressure on the virus could therefore be responsible for the mutation or conservation of the genome of certain traditional proteins or could disturb the expression of certain locations on HIV-1’s genome, influencing ARF expression and escape from ARF CTL responses.

In this study, we evaluated the influence of HAART on HIV-1 ARF T cell responses over the course of HIV-1 infection. We identified new cryptic epitopes derived from sense and antisense transcription of HIV-1. To evaluate the effect of HAART on the ARF T cell responses, we tested PBMCs from HIV-1 acutely infected patients enrolled in a HAART interruption program and HIV-1 chronically infected patients before and after HAART introduction. We report that ARF responses were more frequent and stronger in magnitude in chronically infected individuals compared to acutely infected patients, and importantly that HAART increased the breadth of the ARF responses. Our results indicate that CE could potentially be used to increase the breadth of an HIV-1 vaccine response.

## Materials and Methods

### Subjects

All subjects were recruited at the University of California, San Francisco (UCSF), and were at least 18 years of age at the time of sample collection. All samples were obtained according to protocols approved by the Institutional Review Board (IRB) at UCSF. Written informed consent was obtained from all subjects, according to the Declaration of Helsinki. In order to study the effect of HAART on ARF responses, IFN-γ ELISPOT assays were conducted in 2 different cohorts: the “OPTIONS cohort” with acutely infected individuals, and the “SCOPE cohort” with chronically infected individuals.

The OPTIONS cohort consists of subjects in acute or early HIV-1 infection, with approximately 90% enrolled within 6 months of acquiring HIV-1 infection. For the purposes of this manuscript, subjects in this cohort as designated as “acute” or “acutely infected” to help distinguish them from chronically infected subjects. Cryopreserved PBMC samples were taken from subjects enrolled in the OPTIONS cohort who had undergone a treatment interruption study. To qualify for the treatment interruption (TI) study, participants must have initiated antiretroviral therapy within 6 months of HIV-1 seroconversion, received treatment for at least 24 weeks, and maintained viral loads (VL) below 75 copies/mL for at least 8 weeks prior to entering the protocol. The treatment interruption (TI) protocol was approved by the UCSF IRB, and was designed for patients who initiated antiretroviral therapy in early HIV-1 infection. Under this protocol, treatment would be re-initiated if viral load exceeded certain thresholds (>200,000 copies/mL at any time or >50,000 copies/mL between weeks 4 and 7 of TI). PBMC from each individual was assayed at an early time-point on HAART, with undetectable viral load, and at a later time-point after HAART interruption, with high viral load. Out of 28 acute patients, we tested the 2 time-points (before and after HAART interruption) for 25 patients. We were unable to follow 3 patients at the time-point before HAART interruption.

HIV-1-infected adults were sampled from the Study of the Consequences of the Protease Inhibitor Era (SCOPE), a clinic-based cohort of over 1000 chronically HIV-1-infected individuals at the University of California San Francisco. Chronically infected individuals were tested at an early time-point before HAART treatment with high viral load, and at a later time-point on HAART with undetectable viral load. Out of 21 chronic patients, 18 patients were tested before and after HAART introduction. For 2 patients we only tested the sample before HAART introduction and for 1 patient we only tested the sample after HAART introduction. A description of the cohorts used is depicted in Table 1. Table S1 depicts the viral load, CD4^+^ and CD8^+^ T cell counts for each patient in each group.

**Table 1:** Patients’ characteristics.

Patient group	Number ofpatients	Number ofadditionalpatients	Total number oftested samples	Mean CD4^+^ T cell count (cells/mm^3^)	Mean CD8^+^ T cellcount (cells/mm^3^)	Mean HIV-1 plasmaviral load (copies/mL)
Acute On HAART EarlyTime-point	25	0	25	771	733	59
Acute Off HAART LaterTime-point	25	3	28	625	999	117,000
Chronic Before HAARTEarly Time-point	18	2	20	328	1,439	545,000
Chronic On HAARTLater Time-point	18	1	19	482	1,227	52

Table depicts whether patients were HIV-1 acutely or chronically infected, stage of treatment, the number of patients used in the study, the number of extra patients included in the study, total number of samples tested, mean frequency of CD4^+^ and CD8^+^ T cells (cells/µL) and viral load (copies/mL).

HIV-1 negative, healthy blood donors provided PBMC for control assays (N = 12). No positive CE responses were noted in any healthy blood donor.

### *In silico* Detection of Alternate Reading Frames

Geneious Pro software was used to identify alternative open-reading frames (ORF) starting with an AUG codon in HIV-1 HXB-2 strain. Both forward and reverse ORFs were identified. Forward ORFs with significant similarity to known HIV proteins were eliminated based on batch BLAST searches with manual editing. Out of the 82 forward ORFs identified, 13 did not have significant similarity via BLAST to known HIV proteins. This resulted in the selection of 13 forward ORFs and 70 reverse ORFs for further analysis (Table S2). In the BLAST searches for the 13 selected forward ORFs, 12 generated short hits against small numbers of circulating viral sequence accessions in the NCBI nr protein database (Table S3). These hits did not prompt rejection of the ORF from further consideration, because of low bit scores, and high e-values relative to those ORFs that represented canonical coding sequence for the virus. For these hits, the geographic origin of the sequence, location within the HIV-1 genome, and any data pertinent to the mechanism by which this aberrant sequence may have arisen were recorded. We believe these sequences to be examples of the incorporation of ARF regions into circulating viral sequences isolated from HIV-1 positive subjects.

### *In silico* Peptide Prediction

*In silico* T cell immunogenicity prediction methods were used to identify 9-mer peptide epitopes with potential to be processed by cells, transported into the ER by TAP and bind to HLA molecules in the HLA-B58, A2, and B7 super-families. Peptides were identified within the 13 selected forward and 70 reverse HIV-1 alternate reading frames with NetCTL 1.2 software (http://www.cbs.dtu.dk/services/NetCTL/). All forward ORFs were batched and submitted together to prioritize the highest scoring peptides regardless of individual ORF origin. Top-scoring forward ORF epitopes (N = 22) for HLA-B58, A2, and B7 super-types were selected for peptide synthesis.

Reverse ORFs peptides were also submitted for batch scoring and were prioritized by combining the NetCTL score with additional data regarding previous evidence of ORF expression, ORF length, amino acid similarity to ARF epitopes detected in SIV, and proximity to the 3′ LTR.

Following peptide selection, candidate peptides were again searched using BLAST against a custom database consisting of HXB-2 and consensus B HIV-1 protein sequences gathered from the Los Alamos National Lab HIV sequence databases. Peptides from both forward and reverse reading frame ORFs were included in these searches because any similarity that we expected to detect would have resulted from spurious matches of amino acid sequences within peptides. Peptides were classified according to the numbers of amino acids in common with an HXB-2 and/or consensus B amino acid sequence. Peptides with 5 or fewer amino acids in common were classified as not significantly similar for the purposes of T cell recognition. Peptides with 6 or more amino acids in common are reported.

**Figure 1:**
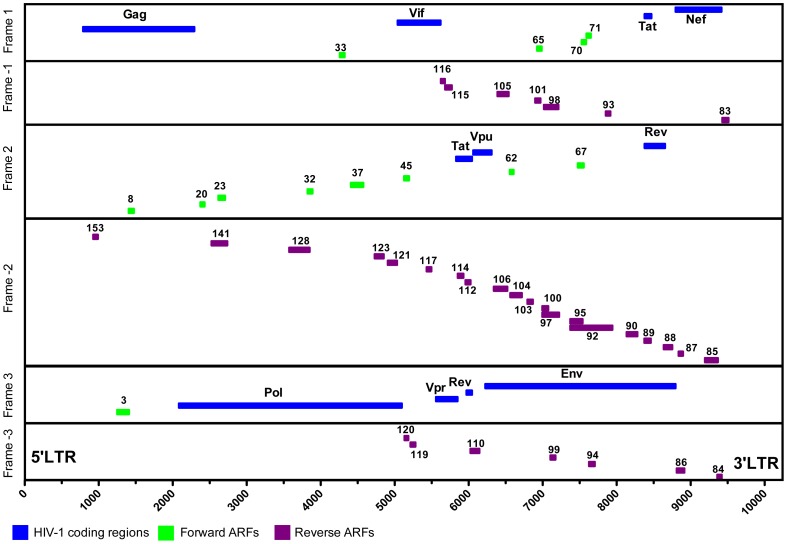
HIV-1 genome (HXB-2 strain), and localization of the 47 alternative reading frames (ARF). Figure depicts all 199 ARF-tested peptides. These include 13 forward ARF within frames 1, 2 or 3 (in green) and 34 reverse ARF within frames -1, -2 or -3 (in purple). HIV-1 classically defined encoding genes are shown in blue.

199 ARF peptides (forward and reverse ORF) were tested: 22 peptides from 13 identified forward ORF (6 predicted HLA-A2, 5 predicted HLA-B7, and 11 predicted HLA-B58 super-type epitopes), the 2 Tat and Rev splice variant peptides previously published [32], and 175 reverse ORF peptides from 34 identified reverse ORF (80 predicted HLA-A2, 38 predicted HLA-B7, and 60 predicted HLA-B58 super-type epitopes) (Figure 1).

### Peptide Synthesis

Peptides were synthesized on NEP array plates (96 wells plates), with 75% purity average at a 2.5 mmol scale with a mass spectrometry analysis of 5 peptides per plate to ensure successful synthesis and positive identification. To evaluate classic HIV-1 T cell responses, a pool of peptides from HIV-1 Gag p24 full protein was used. Table S4 lists the sequences of the ARF individual peptides used in this study and Table S5 lists the content of each ARF pool tested.

### IFN-γ ELISPOT Assays

Immune responses were measured by IFN-γ ELISPOT, as previously described [38]. Data represent the average of two replicate wells minus the average of all negative (no peptide) wells, and are reported as spot-forming cells (SFCs) per million PBMCs, with 100,000 cells added per well. Responses were considered positive if the SFC count was greater than 5 spots (50 SFC per million PBMCs) and greater than twice the background. Peptides were tested at 5 µM/well.

To minimize variability, the same individual ran all experiments and the two time-points from the same patient (“on” versus “off” HAART) were tested on the same day.

### Statistical Analysis

Statistical comparisons were performed using repeated measures logistic regression models. The fixed effects parameters in the model were indicators of “On” versus “Off HAART”, “Acute” versus “Chronic” patients, and the interaction of these two factors. Random effects were used for both study subject and for peptide pool to account for the intra-response correlations. Analyses were performed using Stata 12.

## Results

### ARF Sequences are Found in Viruses with Gross Deletions or Originating in Geographic Regions with a High Prevalence of Recombinants

**Figure 2:**
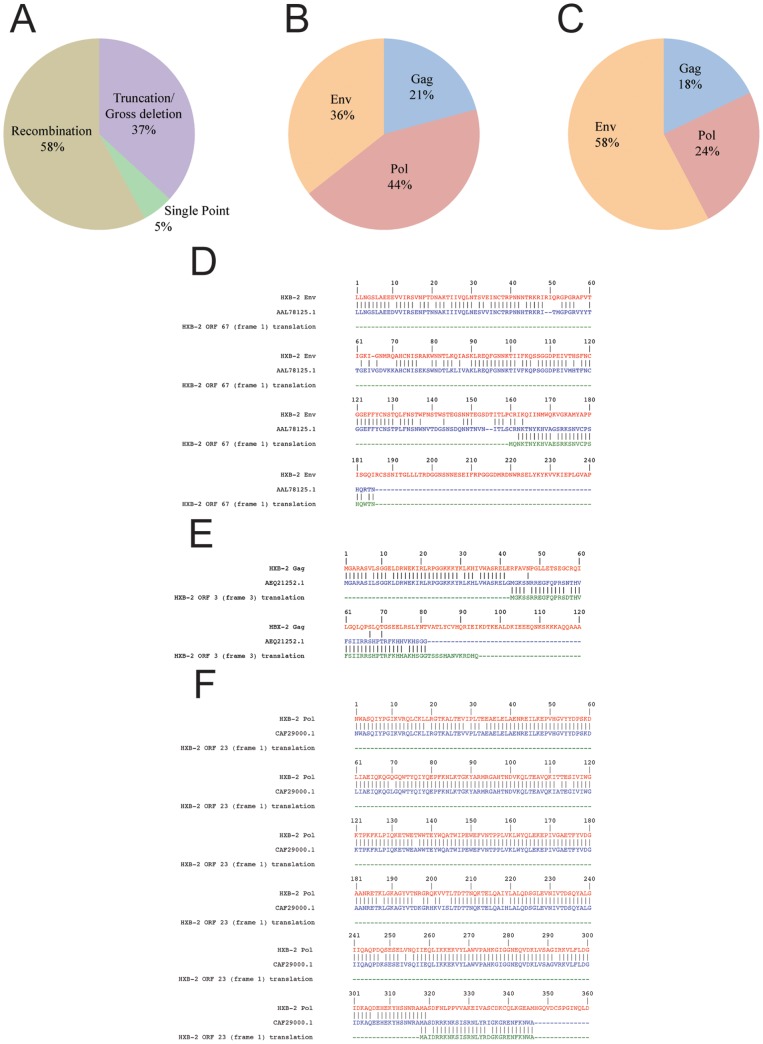
Alternate reading frame-encoded amino acids in circulating viral sequences. A) Distribution of known mutant origins across viral ARF sequences attributable to a particular cause based on information associated with the sequence accession the NCBI nr protein database presented in Table S2. B) Composition of viral coding sequence computed as a percentage assigned to the coding sequence for the Env, Gag, Pol poly-proteins based on nucleotide base counts for a particular gene region compared to the total nucleotide count for the structural genes of the virus. Gag comprises 1,503 nt of the total of 6,878 nt of structural gene sequence; Pol comprises 3,139 nt of the total; Env comprises of 2,571 nt of the total. This is the distribution of origins within the genome that would be expected if originating events for the incorporation of ARF and their detection in circulating HIV-1 viral sequences were distributed randomly throughout the genome. C) The distribution of ARF incorporated into circulating viral sequences that was observed in our searches of NCBI nr protein database for ARF sequences in circulating HIV-1 viral sequences. The percentages were computed by dividing the number of BLAST hits with ARF sequence incorporated into a given gene region by the 123 total hits examined. D) A three-way alignment between the HXB-2 reference sequence for the Env region, the accession AAL78125.1 and the alternate reading frame encoded ORF 67. E) A three-way alignment between the HXB-2 reference sequence for the Gag region, the accession AEQ21252.1 and the alternate reading frame encoded ORF 3. F) A three-way alignment between the HXB-2 reference sequence for the Pol region, the accession CAF29000.1 and the alternate reading frame encoded ORF 23. All three-way alignments were generated by combining two pair-wise alignments created in Geneious, followed by manual editing. Note each accession is similar to both the HXB-2 reference sequence for the structural proteins and the alternate reading frame encoded sequence, but not to both sequences simultaneously within the same region of the sequence.

We found relatively small numbers of short matches with HIV-1 sequences while eliminating candidate ARF regions for structural and accessory proteins. Matches represented circumstances where ARF amino acid sequences were incorporated into predicted sequences for viral proteins. Alternatively, these sequences may possibly represent sequencing errors, indels erroneously introduced into the reported nucleic acid sequence, resulting in a frameshift mutation downstream. We noted the geographic origin, the region of the HIV-1 genome in which the hit was found, gross deletions, circulating recombinant forms or other major features of the sequence to determine causative factors associated with the inclusion of ARF regions in HIV-1 sequences. No mechanism for the incorporation of the ARF was discernable for the majority of ARF sequences detected (70%, referenced in Table S2). Amongst sequences with an attributable origin, over half were associated with either a recombination event or the presence of circulating recombinant forms (Figure 2A). ARFs were also associated with gross deletions, truncations, and point mutations (Figure 2A).

### ARF Sequences are Skewed in Distribution Predominantly in the HIV-1 Env Nucleotide Region

We investigated the genomic region that served as the source of ARF sequences. If incorporation of ARF sequences were a purely random event, the frequency of incorporation would be expected to be proportional to the contribution of each region to the total coding nucleotide sequence for structural proteins of the virus particle (Figure 2B). We determined that the major contributor of ARF sequences was from the HIV-1 env nucleotide region (Figure 1), despite the fact that the env nucleotide region makes only the second largest contribution to the coding sequence as a percentage of the total of coding bases (36% of the coding sequence: 2,751 of 6,878 total coding nucleotides), (Figures 2B and 2C). Interestingly, the HIV-1 pol nucleotide region is responsible for 24% of the total circulating ARF sequences despite its contribution of 44% of the coding sequence in the viral genome (Figures 2B and 2C). To examine more precisely the nature of the similarity between the HXB-2 reference, the ARF region and circulating viral sequences, we created amino acid alignments for a subset of matching accessions. We aligned one matching accession from each region of the genome: the envelope region (Figure 2D), the gag region (Figure 2E) and the pol region (Figure 2F). In each region, amino acid alignments showed similarity both to the HXB-2 reference sequence and to the alternate reading frame sequence, but within distinct regions of each matching sequence (Figures 2D–2F). These data indicate that the viral sequences identified in these accessions are likely hybrids or chimaeras of standard and alternate reading frame encoded sequences.

### Peptides Originating from ARF Regions are Distinguishable in Amino Acid Sequence from Peptides Derived from HIV-1 Structural and Accessory Proteins

We searched our candidate ARF 9-mer peptides for similarity to HIV-1 structural and accessory proteins. None of the ARF peptides were identical in amino acid sequence to any known structural or accessory protein regions of HIV-1. All ARF-derived peptides differed by two or more amino acids from their closest match in a structural or accessory protein of HIV-1. B7ORF70ML9, the peptide that matched at 7 of 9 amino acids was derived from ORF 70 and was a part of Pool #2. There were also four peptides that matched at 6 of 9 amino acids (B7ORF70LV9, B7ORF88SL9, A2ORF97LV9 and A2ORF97CI9 were derived from ORF 70, ORF 88 and ORF 97, respectively). ORF 70 is a Forward ORF, ORFs 88 and 97 are Reverse ORFs. The ORF 88 peptides were a part of Pool #13. The ORF 97 peptides were a part of Pool #15. As a forward reading frame ARF, ORF 70 was included in our searches of the BLAST databases for ARF incorporation (Table S3). There were 5 hits against this ORF, indicating that ORF 70 is detectably incorporated into circulating viral sequences. The two peptides with BLAST hits within this ARF are similar to Vif amino acids 162–169, which is a previously identified HLA-B7 restricted epitope in the Los Alamos database. However, significant similarity does not extend outside this small region, so it does not represent a failure to exclude the first exon of Vif from our tests.

### HIV-1 Acutely Infected Individuals on HAART Mount Detectable Responses Against ARF Pool Peptides

**Figure 3:**
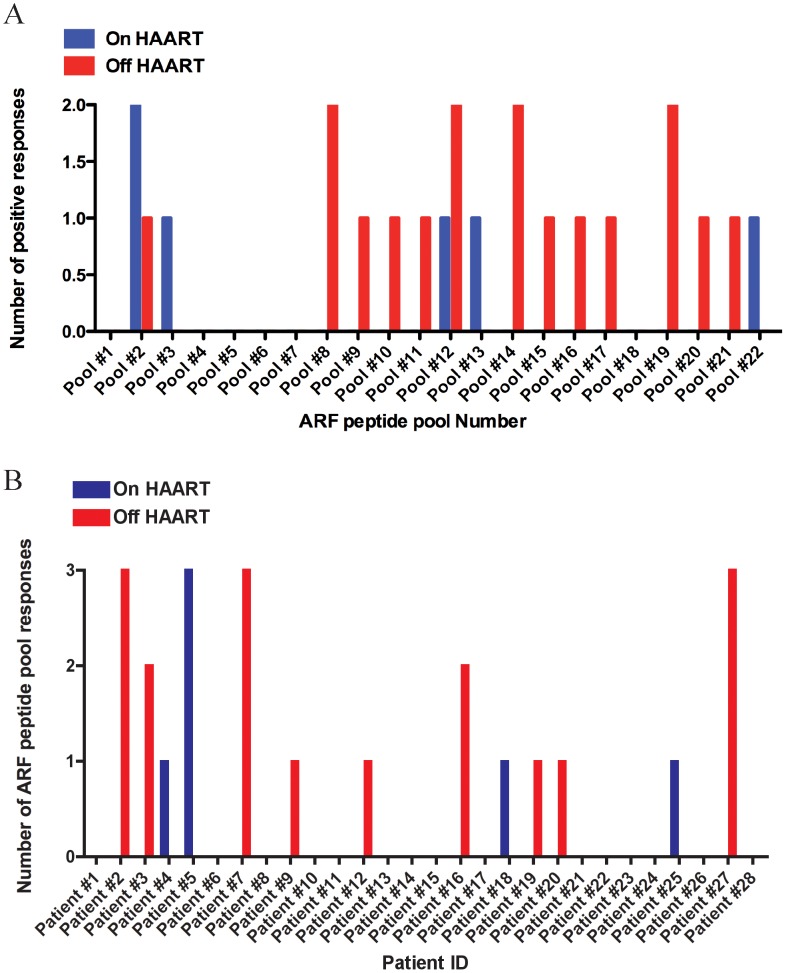
Breadth of ARF responses in acutely infected patients. A) Number of detectable responses observed for each individual ARF peptide-pool tested. B) Number of ARF peptide pools that induced detectable responses in each acutely infected individual. Blue bars represent patients On HAART and red bars represent patients Off HAART. We were unable to follow patients #26, #27 and #28 before HAART interruption.

To assess if ARF T cell responses occur early after HIV-1 infection, we tested PBMC from HIV-1 acutely infected individuals with ARF peptides, and detected the production of IFN-γ in an ELISPOT assay. In order to show the breadth of the responses, Figure 3A depicts the number of detectable responses against the ARF pool peptides tested in patients “On HAART” (blue bars) and “Off HAART” (red bars). For acute subjects, there were a total of 6 responses “On HAART” (1.1% of all wells) versus 17 responses “Off HAART” (2.8%) (p = 0.06 by repeated measures logistic regression). We detected 3 independent responses against ARF peptide pools #2 and #12, followed by pools #8, #14 and #19. Most peptide pools only elucidated one detectable response. It is not possible to discern whether the responses seen in pools 2, 8,12, 14, and 19 in Figure 3A are due to cryptic epitopes or to epitopes with significant homology to the main HIV-1 proteins (peptides P2–407, P2–410; P8–462; P12–490, P12–493; P14–515, P19–569). Figure 3B illustrates the number of ARF peptide pool responses detected in each individual patient tested “On HAART” (blue bars) and “Off HAART” (red bars). Patients #2, #7 and #27 mounted 3 effective ARF responses while off HAART and, in contrast, in patient #5, we did not detect any responses while off HAART, but detected 3 while on HAART. Out of the 25 patients with undetectable VL and at an early time-point after infection, 4 mounted a detectable response against ARF peptides (patients #4, #5, #18 and #25) with an average of 129 SFU (Figure 3). We also determined effective T cell responses against HIV-1 Gag p24 peptides and found it to be on average 315 SFU on 12% of responders (3/25) (data not shown).

### HAART Interruption is Associated with an Increase in Frequency and Magnitude of ARF Responses

**Figure 4:**
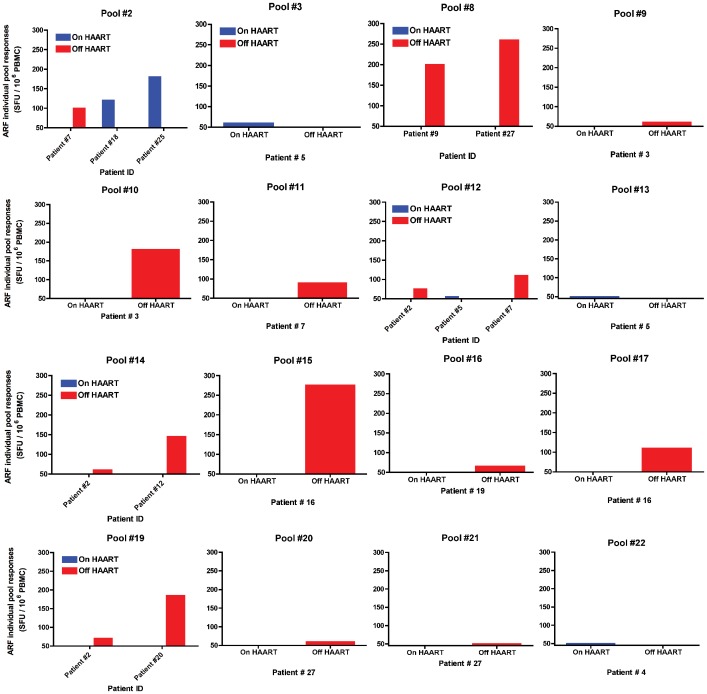
Overview of ARF responses in acutely infected patients. Out of 22 ARF peptide pools tested, 16 induced detectable responses. Each individual graph depicts the intensity of responses measured in an ELISPOT assay against IFN-γ (SFU/million PBMC) for each peptide in the corresponding patient. Blue bars correspond to patients On HAART and red bars to patients Off HAART. Responders were only considered if the net response against the peptide pool was >50 SFU (cut-off) over background and greater than twice the background.

To evaluate whether HAART could play a role in impacting ARF responses in acutely infected patients, we tested a cohort of 28 patients enrolled in a controlled HAART interruption program. Early time-points before HAART interruption were initially tested in 25 of these patients. Figure 4 illustrates the detectable responses against the different ARF pool peptides in patients while On HAART (blue bars), or Off HAART (red bars). Analysis of responses at later time-points after HAART interruption and with high VL revealed 9 out of 28 patients (32% responders) mounted an effective response against the ARF peptide pools with an average of 233 SFU. 14 out of 28 patients (50% responders) had an effective response against HIV-1 Gag p24 peptides with an average response of 777 SFU (data not shown). The data demonstrate that acutely infected patients mount responses against ARF peptides. Overall, 13 out of 28 patients tested (46%) show responses against one or several ARF pools. In this cohort study, patients were enrolled in a controlled HAART interruption program, and responses are rare in patients on HAART (16%), but increase after the interruption of therapy (32%). In addition, the magnitude of ARF responses increased with the interruption of HAART (128 SFU in patients on HAART to 233 SFU in patients off HAART). Interestingly, the dynamics of ARF responses also changed before and after HAART interruption. In this regard, we report that all responses against ARF peptides in patients on HAART were not maintained after the interruption of the treatment. On the other hand, patients on HAART, but with no effective ARF responses were able to mount ARF T cell responses after treatment interruption.

### T Cell Responses Against ARF Pool Peptides are Stronger in HIV-1 Chronically Infected Individuals

**Figure 5:**
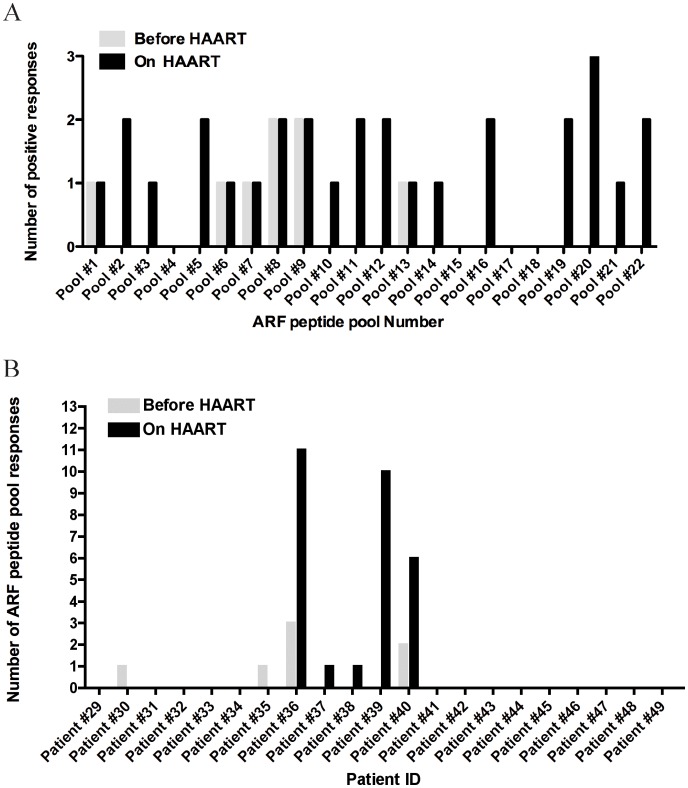
Breadth of ARF responses in chronically infected patients. A) Number of detectable responses observed for each individual ARF peptide-pool tested. B) Number of ARF peptide pools that induced detectable responses in each chronically infected individual. Grey bars represent patients Before HAART and black bars represent patients On HAART. Patients #47 and #48 were only tested before HAART introduction and patient #49 was only tested after HAART.

Next, we investigated whether chronically infected patients could still mount effective responses against ARF pool peptides. For this purpose we tested chronically HIV-1 infected subjects at an early time-point before HAART treatment with high viral load and the same patients at a later time-point while on HAART. In order to show the breadth of the measurable responses, Figure 5A depicts the number of detectable responses against the ARF pool peptides tested in patients “Before HAART” (grey bars) and “On HAART” (black bars). For chronic subjects, there were a total of 29 responses on HAART (6.9% of all wells) versus 8 responses before HAART (1.6%) (p<0.01 by repeated measures logistic regression). In parallel, Figure 5B shows the number of ARF responses detected for each individual chronic patient tested. We found that 20% of the patients tested with high viral load (4/20) responded against ARF pool peptides before the introduction of HAART (Figure 5B). Average ARF responses in this group were 2101 SFU, compared to 1009 SFU against HIV-1 Gag p24 observed in 45% of responders (9/20) (data not shown). Overall, chronically HIV-1 infected patients present stronger and more frequent responses against several ARF pools compared to acutely HIV-1 infected. This suggests that ARF responses increase with the duration of infection.

### The Introduction of HAART in Chronically HIV-1 Infected Patients Increases the Breadth of ARF Responses

**Figure 6:**
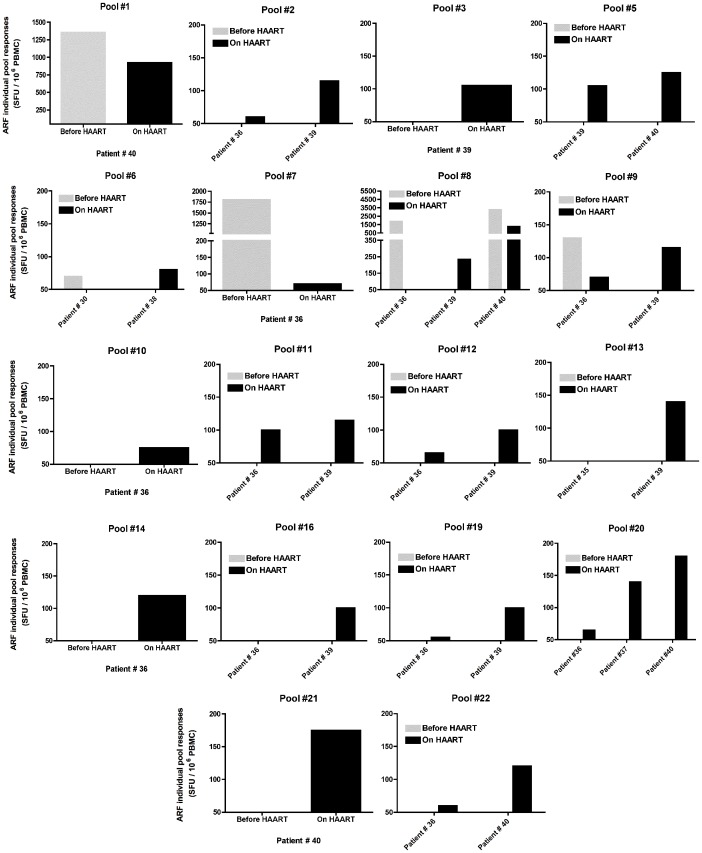
Overview of ARF responses in acutely infected patients. Out of 22 ARF peptide pools tested, 18 induced detectable responses. Each individual graph depicts the intensity of responses measure in an ELISPOT assay against IFN-γ (SFU/million PBMC) for each peptide in the corresponding patient. Grey bars correspond to patients Before HAART and black bars to patients On HAART. Responders were only considered if the net response against the peptide pool was >50 SFU (cut-off) over background and greater than twice the background.

To investigate if HAART had the same impact on ARF responses in chronically HIV-1 infected individuals as it did in acutely infected, we tested the same patients at a later time-point on HAART and with undetectable viral load. We detected effective responses in 26% of the patients tested (5/19) against ARF pool peptides at the later time-point while on HAART with undetectable VL (Figure 6). Although the frequency of responses was higher in patients on HAART, the average magnitude of the responses is lower (978 SFU), compared to the responses against HIV-1 Gag p24 (32% of responders (6/19) with an average response of 658 SFU).

**Figure 7:**
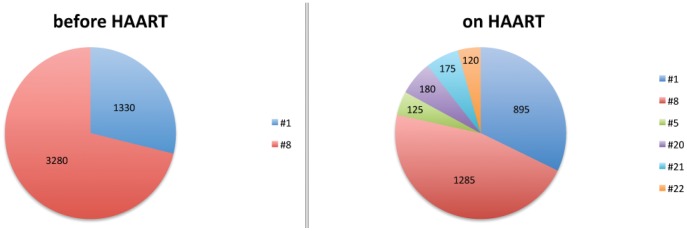
ARF responses in one HIV-1 chronically infected patients before and on HAART. In the pie charts each color represents a pool of immune response that the patient mounted against. Numbers inside the pie charts correspond to the magnitude of the response against the pool in SFU/million PBMC.

Although ARF responses after HAART were diminished in all cases, they still remained at weak to moderate levels, suggesting that cryptic epitopes are still presented, even under suppressive HAART regimens. Overall, we observed a higher number of ARF responses after HAART introduction, suggesting that HAART favors the emergence of new CE responses and increases the breadth of ARF responses during chronic HIV-1 infection. In patient #40, while responses were diminished in magnitude, the number of CE recognized was greater (Figure 7). In this patient before HAART, we detected 2 strong responses against ARF pool peptides #1 and #8. At a later time-point when this patient was on HAART, we were still able to detect the same responses against these 2 pool peptides, but at a lower magnitude. Interestingly, we detected 4 new ARF responses, which were absent before treatment. Responses against alternative spliced variants from TAT or REV (pool #1), previously described in Elite Controllers, were only found in one chronically infected patient, before HAART and on HAART.

## Discussion

In this study, we identified new cryptic epitopes derived from sense and anti-sense transcription of HIV-1. We identified virologic events associated with the incorporation of ARF sequences into circulating viral sequences. Associated events such as recombination, gross deletions and point mutations are consistent with the plausible introduction of frameshift mutations into circulating viral genomes. The origin of the ARF sequences incorporated into coding sequences is noteworthy for the fact that it does not conform to the expectations associated with random distribution of the originating events within the genome. The distribution is not likely to be an effect of each gene region’s total ARF content, as the HIV-1 env nucleotide regions was shown to be the major contributor to known circulating viral ARF sequences, despite containing the smallest percentage of total ARF sequences within its bases. Thus, the env region makes a disproportionally large contribution to the total number of detected ARF sequences compared to its contribution to the total coding sequence. Accessory protein coding regions also exist in this area of the genome, limiting the number of reading frames that could possibly contain ARFs without intersecting with coding sequence for accessory proteins or the env gene itself. The splicing events needed to create some of these accessory proteins may enhance the incorporation of ARF into viral proteins. This novel observation of incorporation of ARF regions into the Env protein could have an impact on the overall plasticity of its amino acid sequence. This plasticity makes a significant contribution to the immune escape of the virus from the neutralizing effects of antibodies targeting Env proteins.

The pol nucleotide region makes a disproportionally small contribution to the total number of ARFs considering the size of this coding region. This may imply constraints that do not permit the incorporation of ARF. As antiretroviral drugs target this region, it is interesting to consider how HAART could modify the dynamics of ARF inclusion in the region.

These circulating, modified viral genomes, together with the translational errors occurring in otherwise intact viral genomes as described previously, are the likely sources for the epitopes that we analyzed in this study. We implemented a number of measures to exclude structural and accessory proteins encoded in standard reading frames of HIV-1 by screening for BLAST similarity at the ORF and peptide levels. None of the ARF-encoded peptides shared more than 7 amino acids in common with any regions of HIV-1 structural or accessory proteins. Five peptides from three of the pools tested shared 6–7 amino acids in common with regions of structural or accessory proteins of HIV-1. While it is important to interpret the immunological results in light of this limited level of similarity, exclusion of these peptides from our analysis was not appropriate. Two of the most similar ARF-encoded peptides originated from a region of alternate reading frame sequence that was detectably incorporated into circulating viral sequences in the NCBI databases. Thus, the immune systems of HIV-1 positive individuals may be exposed to both the structural/accessory-encoded and ARF-encoded variants of these peptides. Additionally, these regions of amino acid similarity may help to elucidate a population of T cells that are potentially cross-reactive between ARF and standard reading frame-encoded peptides.

We evaluated the influence of HAART on HIV-1 ARF T cell responses over the course of HIV-1 infection. In HAART-treated patients, we observed that the frequency and magnitude of T cell responses are higher in chronically infected individuals (26%, 978 SFU) when compared to acutely infected patients (16%, 129 SFU). This suggests that ARF responses increase over the course of HIV-1 infection, and with time, HIV-infected cells have a higher chance of producing and presenting ARF peptides. In chronically HIV-1-infected individuals, HAART decreases the magnitude of ARF responses (from 2100 SFU before to 978 SFU after the introduction of HAART). When ARF responses were analyzed at the individual level, HAART increased the breadth of the responses, which may be due to the selective pressure exerted by drugs on the virus, potentially facilitating the expression of certain ARFs. In acutely infected patients enrolled in a HAART interruption program, we found that HAART interruption favors ARF responses. This could be explained by the fact that HAART exerts a selective pressure on the virus with the emergence of new ARF expression. These immunological results are interesting in light of the relative deficit of ARF incorporation into Pol proteins demonstrated by our database searches. HAART likely alters the dynamics of selective forces acting on the pol nucleotide region and may favor incorporation of some ARF that are otherwise generally disfavored in the region. ARF responses are also present in patients on HAART, even in the absence of detectable anti-HIV-1 T cell responses (from the traditional reading frame), suggesting that ARF products are produced in cells from a latent reservoir. Thus, this therapy or vaccination strategy could specifically target latently infected cells and help in eradication strategies.

### Dynamics of ARF Responses

Although certain pre-existing ARF responses disappeared after the interruption of HAART, new ARF responses emerged. The mechanism underlying ARF expression, mutation and conservation is complex and it involves direct and indirect effects of viral strain and natural evolution within the host, as well as the specifics of the HAART regimen together with patient’s intrinsic characteristics. CTL and HAART both exert a selective pressure on HIV-1 by selecting for CTL or drug escape mutations. The mutational pressure exerted on one location of the HIV-1 genome potentially affects the 3 reading frames, and therefore affects ARFs. ARF T cell responses may therefore be affected through immunological pressure exerted by a classical HIV-1 epitope at the same location, but on a different reading frame. Mutations in one frame do not necessarily lead to the disappearance of an ARF epitope. A non-synonymous change in the one coding frame targeted by the selective pressure may account for a synonymous change in the 2^nd^ or 3^rd^ frames where the ARF epitope is encoded. Therefore, an ARF epitope may be conserved and maintained due to the selective pressure on a different frame. HAART may shape HIV-1′s genome by exerting selective pressure on one or more of the reading frames in a nucleotide region containing an ARF. Therefore, targeting ARF epitopes, especially those that could help the virus escape drug or immune pressure, could provide a new tool to combat viral resistance.

### ARF versus Classic HIV-1 Specific T Cell Responses

In comparison to ARF responses, classically known HIV-1 T cell responses are higher and more frequent in acutely infected individuals. However, in chronically infected patients, the magnitude of ARF responses is similar to the responses to known HIV-1 epitopes. In early infection, the magnitude of ARF responses is below the responses observed for regular CTL HIV responses. We could expect that compared to immune-dominant, known HIV-1 epitopes, ARF peptides would be expressed and presented at a lower density. The similar magnitude of ARF and classic HIV-1 responses in chronically infected subjects could be due to the accumulation with time of the production and presentation of more ARF peptides and/or the viral escape to classic HIV-1 CTL epitopes.

### A Larger Spectrum of ARF Responses

Our data is probably under-estimating the spectrum of ARF responses since we were not able to screen all of the potential peptides encoded within each ARF, but instead used *in silico* predicted epitopes. Because of limitations in cell numbers, we did not test all the selected peptides for immune responses (in particular, many potential antisense ARF identified on the 5′ end of the genome, further from the 3′ LTR). Other ARF without conventional AUG start codons have been described [27], [31], [39] and have not been explored in our present study.

### An ARF Vaccine as a Site-directed HIV Mutagenesis Tool

Taking the phenomenon from a different perspective, the mutational pressure exerted by ARF immune responses could be used to mutate critical proteins of the virus, or to effectively limit the possible mutations permissible in other reading frames. Within the HIV genome, a region that is well conserved due to a high fitness cost for viral protein mutations could potentially contain ARF epitopes in the two other reading frames. Therefore, the immune mutational pressure exerted by an ARF vaccine through CE specific T cells could force the virus to mutate the area containing the ARF epitopes, which would affect the crucial HIV protein encoded in a different frame, rendering the virus defective.

### ARF Responses and Viral Control

The duration of infection and HAART play a role on the magnitude and breadth of ARF responses. As an observational cross sectional study, our data does not show whether ARF responses help in controlling HIV-1 infection. Previous studies have shown that the magnitude of the CD8^+^ T cell response poorly correlates with immune control of HIV-1 infection [10], [37] whereas the breadth of the CTL response seems to be better associated with viral control [50], [51]. Previous *in vitro* studies have shown viral suppression by ARF CTLs [30], and viral escape from an ARF CD8^+^ T cell response has been described in monkey studies, supporting their immunogenicity *in vivo* [30]. Recently, human data suggested that responses to ARF-encoded HIV-1 epitopes contribute to viral control *in vivo,* and drives viral evolution on a population level [26], [27]. We have previously reported more frequent CE responses in patients with well-controlled HIV-1 infection [32].

ARF responses could potentially be used as a diagnostic tool to monitor HIV-1 immunity through T cell responses against ARF peptides, and could be a surrogate marker to measure the ability of an anti-retroviral drug regimen to work against the latent reservoir.

## Supporting Information

Table S1 **Viral load, CD4^+^ and CD8^+^ T cell counts for each individual patient in both the Acute and Chronic groups.**
(XLSX)

Table S2 **Complete description of nucleotide and amino acid sequences used in the study.**
(XLSX)

Table S3 **BLAST searches for the selected forward ORFs.**
(PDF)

Table S4 **ARF individual peptides.** Table lists the number of the ARF peptide, peptide name, peptide sequence and number of pool.
(PDF)

Table S5 **ARF pooled peptides.** Table lists the number of peptide pool, the pool content and the total number of peptides in the pool.
(PDF)
