# Supplementary material for: Influence of HAART on Alternative Reading Frame Immune Responses over the Course of HIV-1 Infection
Source: PLoS One. 2012 Jun 29;7(6):e39311. doi: 10.1371/journal.pone.0039311 (PMC3387156; doi:10.1371/journal.pone.0039311)
Supplement: Table S3 — BLAST searches for the selected forward ORFs. (PDF) [file pone.0039311.s003.pdf]

Expressed Gag ARF Search Results

ARF 3 (Frame 3)

Accession#	Mechanism	Title	Bit Score	e Value	Query Coverage	Max Identity
AEQ21252	Gross Deletion	Association of high frequency of gross deletions in the pol and 5'LTR/gag genes with Korean red ginseng intake in HIV-1 infected	77.4	2.00E-18	75%	92%
CAC05270	Recom	Recombinant strains of HIV type 1 in the United Kingdom	77.4	4.00E-17	90%	77%
AAP84333	Trunc	Fine specificity and cross-clade reactivity of HIV type 1 Gag-specific CD4+ T cells	53.1	3.00E-09	96%	54%
AAC40703	Other	Molecular evidence for nosocomial transmission of human immunodeficiency virus from a surgeon to one of his patients	50.8	4.00E-07	88%	50%
CAE54686	Recom	Development, evaluation, and validation of an oligonucleotide probe hybridization assay to subtype human immunodeficiency virus type 1	42	2.00E-04	63%	61%
AAF28557	Recom	Simplified strategy for detection of recombinant humanimmunodeficiency virus type 1 group M isolates by gag/env heteroduplex mobility assay. Study Group on Heterogeneity of HIV Epidemics in African Cities	43.1	3.00E-04	61%	63%
CAF74739	Other	Genetic subtyping of gag and env regions of HIV type 1 isolates in Republic of Congo	39.7	0.005	88%	43%
CAC05266	Recom	Recombinant strains of HIV type 1 in the United Kingdom	37	0.038	88%	37%

AAY84633	Trunc	HIV-1 specific CD4+ T cell responses are not associated with significant viral epitope variation in persons with persistent plasma viremia	35.8	0.041	96%	38%
ABU42059	Gross Deletion	High frequency of gross deletions in the 5' LTR and gag regions in HIV type 1-infected long-term survivors treated with Korean red ginseng	36.6	0.071	71%	49%
AAF28621	Recom	Simplified strategy for detection of recombinant human immunodeficiency virus type 1 group M isolates by gag/env heteroduplex mobility assay. Study Group on Heterogeneity of HIV Epidemics in African Cities.	36.2	0.1	44%	65%
ABI80019	Other	Selection on the human immunodeficiency virus type 1 proteome following primary infection	33.5	0.74	28%	100%
CAF74725	Other	Genetic subtyping of gag and env regions of HIV type 1 isolates in	32.7	1.5	88%	37%
CAA09506	Other	HIV type 2 pathogenicity is not related to subtype in rural Guinea Bissau	31.2	4.2	88%	37%

ARF 8 (Frame 2)

**Accession#**	**Mechanism**	**Title**	**Bit Score**	**e Value**	**Query Coverage**	**Max Identity**
AAB49839	Recom	Human immunodeficiency virus type 1 subtypes defined by env show high frequency of recombinant gag genes. The UNAIDS Network for HIV Isolation and Characterization	44.8	1.00E-04	54%	100%
CAA82791	Other	Molecular epidemiology of HIV-1 in Madrid	32	2.9	36%	100%
CAA82789	Other	Molecular epidemiology of HIV-1 in Madrid	32	2.9	36%	100%

CAA82792	Other	Molecular epidemiology of HIV-1 in Madrid	32	2.9	36%	100%
CAA82790	Other	Molecular epidemiology of HIV-1 in Madrid	32	2.9	36%	100%
CAA82788	Other	Molecular epidemiology of HIV-1 in Madrid	32	2.9	36%	100%
CAA82796	Other	Molecular epidemiology of HIV-1 in Madrid	32	2.9	36%	100%
CAC05266	Recom	Recombinant strains of HIV type 1 in the United Kingdom	27.4	124	77%	59%

Expressed Pol ARF Search Results

ARF 20 (Frame 2)

**Accession#**	**Mechanism**	**Title**	**Bit Score**	**e Value**	**Query Coverage**	**Max Identity**
AAY29809	Trunc	A Novel Assay Allows Genotyping of the Latent Reservoir for Human Immunodeficiency Virus Type 1 in the Resting CD4+ T Cells of Viremic Patients	51	3.00E-07	77%	100%
AAL85844	Other	Demonstration of de Novo HIV Type 1 Production by Detection of Multiply Spliced and Unspliced HIV Type 1 RNA in Paraffin-	29.9	8.7	44%	100%

ARF-23 (Frame 2)

**Accession#**	**Mechanism**	**Title**	**Bit Score**	**e Value**	**Query Coverage**	**Max Identity**
CAF29000	Other	Antiviral activity of reverse trancriptase inhibitors with	86.3	8.00E-19	100%	93%
AAZ91644	Other	Genomic Diversity of HIV-1 subtypes in Northern Kenya	78.3	5.00E-16	96%	82%
AAZ91642	Other	Genomic Diversity of HIV-1 subtypes in Northern Kenya	77.4	1.00E-15	100%	86%
AAS79329	Other	Documentation of subtype C HIV Type 1 strains in Argentina,	63	1.00E-10	89%	77%

AAN40299	Other	HIV-1 subtypes in Luxembourg, 1983-2000	41.8	0.003	62%	64%
AAZ91645	Trunc	Genomic Diversity of HIV-1 subtypes in Northern Kenya	41.4	0.004	62%	72%
AAU13857	Other	Molecular Epidemiology of HIV-1 in Thailand	38	0.054	41%	92%
AAL70819	Other	Multiple viral genetic analyses detect low-level human immunodeficiency virus type 1 replication during effective highly active antiretroviral therapy	35.4	0.44	37%	91%
AEL30637	Other	Genetic diversity and drug resistance profiles in HIV-1 infected patients from Cape Verde Islands	33.3	2.4	37%	82%
AAS76977	Other	Overview of genotypic and clinical profiles of human immunodeficiency virus type 1-infected children in Rio de Janeiro, Brazil	32.9	3.3	34%	90%
ABB89863	Single Point	Comparative selection of the K65R and M184V/I mutations in human immunodeficiency virus type 1-infected patients enrolled in a trial	32	6.5	41%	75%
ABU89197	Recom	Highly divergent subtypes and new recombinant forms prevail in the HIV/AIDS epidemic in Angola: new insights into the origins of the Aids pandemic	32	6.7	68%	52%
AAQ74952	Other	Molecular epidemiologic study of a human immunodeficiency virus 1	29.9	35	48%	57%

ARF 32 (Frame 2)

Accession#	Mechanism	Title	Bit Score	e Value	Query Coverage	Max Identity
ADZ33585	Other	Direct Submission	68.9	3.00E-13	100%	95%
CAC86176	Other	Resistance to antiretroviral treatment in Gabon: need for implementation of guidelines on antiretroviral therapy use and HIV-1 drug resistance monitoring in developing countries	44.8	9.00E-05	66%	93%
AAY25729	Other	Molecular epidemiology of HIV Type 1 in preparation for a Phase III prime-boost vaccine trial in Thailand and a new approach to HIV Type 1 genotyping	41.4	0.001	71%	80%

ARF 33 (Frame 1)

Accession#	Mechanism	Title	Bit Score	e Value	Query Coverage	Max Identity
ADP94121	Other	Analysis of pol integrase gene sequences of HIV-1 infected treatment naive individuals in the Limpopo province of South Africa	45.6	1.00E-05	76%	81%

ARF 37 (Frame 2)

Accession#	Mechanism	Title	Bit Score	e Value	Query Coverage	Max Identity
AAY57410	Trunc	Genetic characterization of full-length HIV type 1 genomes from 3 infected paid blood donors in Henan, China	75.9	1.00E-15	74%	83%
AAZ94952	Other	Genetic Diversity of HIV-1 in Northern Kenya	45.1	2.00E-05	54%	77%
CAA08123	Other	HIV type 2 pathogenicity is not related to subtype in rural Guinea Bissau	45.4	2.00E-05	98%	44%
CAA08092	Other	HIV type 2 pathogenicity is not related to subtype in rural Guinea Bissau	43.9	9.00E-05	98%	63%

AAZ94959	Other	Genetic Diversity of HIV-1 in Northern Kenya	36.6	0.043	43%	75%
CAA08033	Other	HIV type 2 pathogenicity is not related to subtype in rural Guinea Bissau	35.4	0.14	98%	35%
AAZ94969	Other	Genetic Diversity of HIV-1 in Northern Kenya	35	0.16	61%	53%
ABD52825	Other	HIV Type 1 Subtypes among STI Patients in Nairobi: A Genotypic Study Based on Partial pol Gene Sequencing	33.9	0.37	34%	79%
AAZ94981	Other	Genetic Diversity of HIV-1 in Northern Kenya	32.3	1.6	43%	67%
ABD52822	Other	HIV Type 1 Subtypes among STI Patients in Nairobi: A Genotypic Study Based on Partial pol Gene Sequencing	31.6	2.8	34%	79%
ABD52821	Other	HIV Type 1 Subtypes among STI Patients in Nairobi: A Genotypic Study Based on Partial pol Gene Sequencing	30.8	5.6	38%	67%

Expressed Env ARF Search Results

ARF 62 (Frame 2)

**Accession#**	**Mechanism**	**Title**	**Bit Score**	**e Value**	**Query Coverage**	**Max Identity**
ADA84563	Other	Characterization of emergent HIV resistance in treatment-naive subjects enrolled in a vicriviroc phase 2 trial	41.4	6.00E-04	100%	81%
AAB05195	Other	HIV type 1 variation in World Health Organization-sponsored vaccine evaluation sites: genetic screening, sequence analysis, and preliminary biological characterization of selected viral strains. WHO Network for HIV Isolation and Characterization	38.8	0.005	93%	73%

AAK77505	Other	Genetic analysis of the complete gag and env genes of HIV type 1 subtype C primary isolates from South Africa	35.8	0.052	68%	82%
ADP37054	Other	Direct Submission	35	0.1	68%	82%
ADA84557	Other	Characterization of emergent HIV resistance in treatment-naïve subjects enrolled in a vicriviroc phase 2 trial	32	1.2	81%	77%
ACS91274	Recom/Trunc	Longitudinal quasispecies analysis of viral variants in HIV type 1dually infected individuals highlights the importance of sequence identity in viral recombination	30.3	4.7	68%	73%
AAL11074	Recom	Human immunodeficiency virus type 1 clade A and D neurotropism: molecular evolution, recombination, and coreceptor use	30.3	4.8	56%	89%

ARF-65 (Frame 1)

**Accession#**	**Mechanism**	**Title**	**Bit Score**	**e Value**	**Query Coverage**	**Max Identity**
AAB61425	Other	Development of AIDS in a chimpanzee infected with human immunodeficiency virus type 1	57.5	2.00E-09	90%	89%
AEB00525	Other	Independent evolution of variable and constant regions of human immunodeficiency virus type 1 (HIV-1) env glycoprotein gp120	47.3	9.00E-06	85%	82%
ADA84548	Other	Characterization of emergent HIV resistance in treatment-naïve subjects enrolled in a vicriviroc phase 2 trial	45.6	4.00E-05	70%	86%
ACT53938	Recom	Longitudinal quasispecies analysis of viral variants in HIV type 1 dually infected individuals highlights the importance of sequence identity in viral recombination	43.9	1.00E-04	65%	92%

CAA12240	Other	The subtypes of HIV type 1 in Greece	43.5	2.00E-04	70%	86%
ABS89899	Other	Characteristics of HIV type 1 (HIV-1) glycoprotein 120 env sequences in mother-infant pairs infected with HIV-1 subtype CRF01_AE	43.5	2.00E-04	85%	71%
AAC33082	Recom	HIV-1 genetic subtype A/B recombinant strain causing an explosive epidemic in injecting drug users in Kaliningrad	42.2	6.00E-04	60%	92%
ACT54220	Recom	Longitudinal quasispecies analysis of viral variants in HIV type 1 dually infected individuals highlights the importance of sequence identity in viral recombination	32.5	1.6	55%	82%
ACS91252	Recom	Longitudinal quasispecies analysis of viral variants in HIV type 1 dually infected individuals highlights the importance of sequence identity in viral recombination	30.8	6.2	50%	80%
ADP55165	Recom	HIV-1 subtype distribution in the Gambia and the significant presence of CRF49_cpx, a novel circulating recombinant form	27.4	99	50%	80%
ADP55144	Recom	HIV-1 subtype distribution in the Gambia and the significant presence of CRF49_cpx, a novel circulating recombinant form	27.4	99	50%	80%

ARF 67 (Frame 2)

**Accession#**	**Mechanism**	**Title**	**Bit Score**	**e Value**	**Query Coverage**	**Max Identity**
AAL78125	Other	Human immunodeficiency virus type 1 bound to B cells: relationship to virus replicating in CD4+ T cells and circulating in plasma	71.5	8.00E-14	92%	92%
ABI80936	Trunc	Selection on the human immunodeficiency virus type 1 proteome following primary infection	71.5	8.00E-14	92%	92%

AAL29365	Single Point	T69D/N pol mutation, human immunodeficiency virus type 1 RNA levels, and syncytium-inducing phenotype are associated with CD4 cell depletion during didanosine therapy.	68.9	6.00E-13	100%	85%
ACA49454	Other	High frequency of BF mosaic genomes among HIV-1-infected children from Sao Paulo, Brazil	64.7	2.00E-11	100%	81%
AAC05790	Other	Human immunodeficiency virus type 1 subtypes B and C detected in New Zealand	60.9	4.00E-10	84%	82%
AAF28097	Other	The explosive human immunodeficiency virus type 1 epidemic among injecting drug users of Kathmandu, Nepal, is caused by a subtype C virus of restricted genetic diversity.	59.6	1.00E-09	84%	82%
ABI79981	Other	Selection on the human immunodeficiency virus type 1 proteome following primary infection	58.7	2.00E-09	76%	90%
ABI79972	Other	Selection on the human immunodeficiency virus type 1 proteome following primary infection	58.7	2.00E-09	76%	90%
ABI79963	Other	Selection on the human immunodeficiency virus type 1 proteome following primary infection	58.7	2.00E-09	76%	90%
ABI79955	Other	Selection on the human immunodeficiency virus type 1 proteome following primary infection	58.7	2.00E-09	76%	90%
CAJ77398	Recom	HIV-1 subtypes and recombinants in the Republic of Congo	58.3	3.00E-09	84%	86%
AAM46030	Other	HIV type 1 group M clades infecting subjects from rural villages in equatorial rain forests of Cameroon	56.6	1.00E-08	84%	82%

ADE61834	Trunc	4E10-resistant HIV-1 isolated from four subjects with rare membrane-proximal external region polymorphisms	56.6	1.00E-08	84%	77%
CAD87076	Other	Biological and genetic characteristics of HIV infections in Cameroon reveals dual group M and O infections and a correlation between SI-inducing phenotype of the predominant CRF02_AG variant and disease stage.	52.4	4.00E-07	92%	75%
CAC15260	Other	Unprecedented degree of human immunodeficiency virus type 1 (HIV-1) group M genetic diversity in the Democratic Republic of Congo suggests that the HIV-1 pandemic originated in Central Africa	48.6	8.00E-06	84%	73%
ABI80924	Other	Selection on the human immunodeficiency virus type 1 proteome following primary infection	47.7	2.00E-05	65%	88%
AAC58921	Other	Immunological and virological analyses of persons infected by human immunodeficiency virus type 1 while participating in trials of recombinant gp120 subunit vaccines	44.8	2.00E-04	73%	79%
AAX38743	Other	In-depth, longitudinal analysis of viral quasispecies from an individual triply infected with late-stage human immunodeficiency virus type 1, using a multiple PCR primer approach	44.8	2.00E-04	53%	93%
ADZ39073	Trunc	Clonal Sequences Recovered from Plasma from Patients with Residual HIV-1 Viremia and on Intensified Antiretroviral Therapy Are Identical to Replicating Viral RNAs Recovered from Circulating Resting CD4+ T Cells	43.9	3.00E-04	73%	79%

AAU14919	Recom	Identification of a novel HIV-1 complex circulating recombinant form (CRF18_cpx) of Central African origin in Cuba	41.4	0.003	73%	68%
AAK01777	Other	Use of drug resistance sequence data for the systematic detection of non-B human immunodeficiency virus type 1 (HIV-1) subtypes: how to create a sentinel site for monitoring the genetic diversity of HIV-1 at a country scale	40.5	0.005	42%	100%
ACL98565	Other	Genetic characterization of HIV-1 from semen and blood from clade C-infected subjects from India and effect of therapy in these body compartments	40.5	0.005	46%	92%
ABF55180	Other	Emergence of ART resistance associated primary mutations among drug naive HIV-1 individuals in rural western Cameroon	40.1	0.007	50%	92%
AAT07385	Other	Genetic diversity of HIV type 1 in rural eastern Cameroon	39.2	0.015	50%	85%
AAC58924	Recom	Immunological and virological analyses of persons infected by human immunodeficiency virus type 1 while participating in trials of recombinant gp120 subunit vaccines.	39.2	0.015	50%	92%
AAY19639	Other	Transmission of cell-free and cell-associated HIV-1 through breast-feeding	38.4	0.029	57%	80%
AAT07383	Other	Genetic diversity of HIV type 1 in rural eastern Cameroon	38	0.041	61%	75%
ABY71536	Other	Genetic and Neutralization Properties of HIV-1 env Clones from Subtype B/BC/AE Infections in China	37.1	0.083	42%	91%
AAO86401	Other	The pathogenesis of three different human immunodeficiency virustype 1 populations in a triply-infected patient	36.7	0.11	53%	79%

ADA84563	Other	Characterization of emergent HIV resistance in treatment-naïve subjects enrolled in a vicriviroc phase 2 trial	36.7	0.12	57%	80%
AAU85704	Recom	HIV-1 subtype and viral tropism determination for evaluating antiretroviral therapy options: an analysis of archived Kenyan blood samples	33.7	1.2	50%	77%
CAD87251	Other	Biological and genetic characteristics of HIV infections in Cameroon reveals dual group M and O infections and a correlation between SI-inducing phenotype of the predominant CRF02_AG variant and disease stage	32.5	3.4	100%	50%
AAT07360	Other	Genetic diversity of HIV type 1 in rural eastern Cameroon	32	4.7	57%	73%
CAD87059	Other	Biological and genetic characteristics of HIV infections in Cameroon reveals dual group M and O infections and a correlation between SI-inducing phenotype of the predominant CRF02_AG variant and disease stage	32	4.8	53%	71%
CAD87120	Other	Biological and genetic characteristics of HIV infections in Cameroon reveals dual group M and O infections and a correlation between SI-inducing phenotype of the predominant CRF02_AG variant and disease stage	30.8	13	50%	85%
CAJ77397	Recom	HIV-1 subtypes and recombinants in the Republic of Congo	29.5	36	80%	57%
CAD36353	Other	High genetic diversity of HIV-1 strains in Chad, West Central Africa	29.5	36	76%	55%

AAX48987	Other	Molecular Characteristics of HIV Type 1 Circulating in Sao Paulo, Brazil	29.1	51	73%	63%

ARF 70 (Frame 1)

Accession #	Mechanism	Title	Bit Score	e Value	Query Coverage	Max Identity
ABC88325	Recom	Analysis of the near full length genomes of HIV-1 subtypes B, F and BF recombinant from a cohort of 14 patients in Sao Paulo, Brazil	58.3	1.00E-09	100%	89%
AAA97843	Other	Adaptive Evolution of Human Immunodeficiency Virus Type 1 During the Natural Course of Infection	54.9	2.00E-08	100%	89%
AAG15920	Trunc	Human immunodeficiency virus type 1 shedding pattern in semen correlates with the compartmentalization of viral Quasi species between blood and semen	50.7	5.00E-07	84%	94%
AAK66422	Trunc	A Persistent Reservoir for HIV-1 in Alveolar Macrophages	41.8	7.00E-04	84%	81%
ADO24389	Other	Molecular viral factors in Human immunodeficiency virus type-1 mother-to-child transmission: characterization of a group of infected mothers from Oporto	38	0.015	84%	81%
ABW37206	Other	Impact of HIV-1 genetic diversity in China on the measurement of viral load	34.1	0.34	47%	100%
BAE95924	Other	Infectious DNA clones of subtype G and CRF02_AG HIV-1 derived from Ghananian isolates	29.5	15	57%	82%

ARF 71 (Frame 1)

Accession #	Mechanism	Title	Bit Score	e Value	Query Coverage	Max Identity
AAB52796	Other	Analysis of the V1-V5 env region in Long-Term Non Progressor and in rapid progressor HIV-1 infected individuals	35.8	0.073	88%	75%
AAR95931	Other	Evidence that low-level viremias during effective highly active antiretroviral therapy result from two processes: expression of archival virus and replication of virus	35.4	0.1	66%	83%
AAL29458	Trunc	Multiprotein-expressing DNA Component for a DNA/MVA Vaccine for HIV-1	35	0.15	94%	71%
AEP93955	Other	Impact of Mutations Outside the V3 Region on Coreceptor Tropism Phenotypically Assessed in Patients Infected with HIV-1 Subtype B	34.6	0.21	100%	70%
ABC88325	Other	Analysis of the near full length genomes of HIV-1 subtypes B, F and BF recombinant from a cohort of 14 patients in Sao Paulo, Brazil	32.9	0.82	74%	71%
AAR95932	Other	Evidence that low-level viremias during effective highly active antiretroviral therapy result from two processes: expression of archival virus and replication of virus.	31.6	2.3	55%	90%
ADF84309	Trunc	Adaptive interactions between HLA and HIV-1: highly divergent selection imposed by HLA class I molecules with common supertype motifs	29.5	13	88%	63%
AAP59056	Other	Compartmentalization and migration of HIV-1 between peripheral blood and cerebrospinal fluid	29.1	18	88%	61%
