# Supplementary material for: Influence of HAART on Alternative Reading Frame Immune Responses over the Course of HIV-1 Infection
Source: PLoS One. 2012 Jun 29;7(6):e39311. doi: 10.1371/journal.pone.0039311 (PMC3387156; doi:10.1371/journal.pone.0039311)
Supplement: Table S4 — ARF individual peptides. Table lists the number of the ARF peptide, peptide name, peptide sequence and number of pool. (PDF) [file pone.0039311.s004.pdf]

Supplemental Table S4

peptide #	Peptide name	Sequence	POOL #
378	Splice TAT leader	SRDIHHYRFR	1
379	Splice REV leader	RIFTIIVSD	1
400	A2ORF_08KI9	KLQNGIECI	2
401	A2ORF_45KI9	KLGDGFIDI	2
402	A2ORF_62AL9	AMCKINPTL	2
403	A2ORF_65VV9	VQMSAQYNV	2
404	A2ORF_33TV9	TVIGEQWLV	2
405	A2ORF_65AL9	AQYNVHMEL	2
* 406	B7ORF_03QF9	QPRSDTHVF	2
407	B7ORF_70ML9	MPLPSVDKL	2
408	B7ORF_37YQ9	YPGSSSCSQ	2
409	B7ORF_37RF9	RAGNSIFSF	2
410	B7ORF_70LV9	LPSVDKLDV	2
411	B58ORF_37RF9	RAGNSIFSF	3
412	B58ORF_32RW9	RSRNLLCRW	3
413	B58ORF_37SY9	SSSCSQWIY	3
414	B58ORF_65MM9	MSAQYNVHM	3
415	B58ORF_37KY9	KMASKNNTY	3
416	B58ORF_03RI9	RSDTHVFSI	3
417	B58ORF_33NW9	NITVIGEQW	3
418	B58ORF_71RI9	RSSDLEEEI	3
419	B58ORF_20KF9	KNDRGNWRF	3
420	B58ORF_23KW9	KGRENFKNW	3
421	B58ORF_67SW9	SNVCPSHQW	3
422	A2ORF_092YL9	YLYNSLLQL	4
423	A2ORF_092KL9	KISDSLLLL	4
424	A2ORF_092AL9	ALFSLCTTL	4
425	A2ORF_092VL9	VLLNSCVEL	4
426	A2ORF_092HL9	HMFIICFIL	4
427	A2ORF_092TL9	TLYLYNSLL	4
428	A2ORF_092SL9	SLLLLPPSL	4
429	A2ORF_092SL9	SLISPPPGL	4
430	A2ORF_092RV9	RVIVSLPSV	4
431	A2ORF_092SV9	SVIEAAPIV	4
432	A2ORF_092FV9	FILHGRVIV	5
433	A2ORF_092SI9	SLVNSSPVI	5
434	A2ORF_092LV9	LLLLPPSLV	5
435	A2ORF_092TA9	TLLFALVGA	5
436	B7ORF_092AA9	APKNPRNKA	5
437	B7ORF_092FI9	FPTFCHMFI	5
438	B7ORF_092SL9	SPVIFDEHL	5
439	B7ORF_092CI9	CPLMGGAYI	5
440	B7ORF_092IC9	IPDNNCLAC	5
441	B58ORF_092IM9	IAFPTFCHM	6
442	B58ORF_092GF9	GAYIAFPTF	6

* Cardineau et al., 2004 J Exp Med

peptide #	Peptide name	Sequence	POOL #
443	B58ORF_092FF9	FSLCTTLLF	6
444	B58ORF_092LF9	LVNSSPVIF	6
445	B58ORF_092AF9	ASIALSKLF	6
446	B58ORF_092GL9	GSIFTTLYL	6
447	B58ORF_092IF9	IVSLPSVLF	6
448	B58ORF_092KF9	KAPIPTALF	6
449	B58ORF_092LF9	LMGGAYIAF	6
# 450	A2ORF_128SV9	SVNCFTSLV	7
451	A2ORF_128VV9	VVIFCTASV	7
452	A2ORF_128SL9	SLVWAPLIL	7
453	A2ORF_128IV9	IQVACQYSV	7
454	A2ORF_128MT9	MLSVVIFCT	7
455	A2ORF_128GL9	GVFPHITML	7
456	A2ORF_128CV9	CMGSLNLGV	7
457	A2ORF_128PV9	PLILAYFPV	7
458	A2ORF_128LA9	LVWAPLILA	7
459	B7ORF_128FV9	FPHITMLSV	8
460	B7ORF_128HL9	HVSFCMGSL	8
x 461	B58ORF_128LF9	LAYFPVFRF	8
462	B58ORF_128IF9	ITMLSVVIF	8
463	B58ORF_128SY9	SGIQVACQY	8
464	B58ORF_128HM9	HVSHVSFCM	8
465	B58ORF_128VW9	VNCFTSLVW	8
466	B58ORF_128LF9	LILAYFPVF	8
467	A2ORF_141VI9	VLYGFSGPI	9
468	A2ORF_141FL9	FTGTVSIGL	9
469	B7ORF_141SF9	SVQISTNAF	9
470	B7ORF_141GF9	GPSIPGFNF	9
471	B7ORF_141FQ9	FPSFSISVQ	9
472	B58ORF_141FF9	FSGPIFEIF	9
473	B58ORF_141SF9	SVNGHCLTF	9
474	B58ORF_141VF9	VSIGLMGKF	9
475	B58ORF_141QF9	QISTNAFIF	9
476	B58ORF_141FL9	FSSVNGHCL	9
477	A2ORF_153FL9	FVYSLLMFL	10
478	A2ORF_153MV9	MVVAVPVFV	10
479	A2ORF_153AL9	AVPVFVYSL	10
480	B7ORF_153VL9	VPVFVYSLL	10
481	B58ORF_153VY9	VVAVPVFVY	10
482	B7ORF_083SL9	SPVPPRPRL	11
483	B7ORF_083RP9	RPRLPGKSP	11
484	B7ORF_083RP9	RARHSPVPP	11
485	B7ORF_084RM9	RMQLSGHVM	11
486	A2ORF_085LV9	LLSYLAQLV	12
487	A2ORF_085PL9	PLLSYLAQL	12

Bansal et al., 2010 J Exp Med

x Peptide close to AYFPVFRFL, Bansal et al., 2010 J Exp Med

peptide #	***Peptide name***	***Sequence***	**POOL #**
488	A2ORF_085GL9	GVTSWCSLL	12
489	B58ORF_085TW9	TSWCSLLYW	12
490	B58ORF_085LL9	LSYLAQLVL	12
491	B58ORF_085VY9	VTSWCSLLY	12
492	B58ORF_085AW9	AGSQGVTSW	12
493	B58ORF_085LY9	LLYWPLLSY	12
494	B58ORF_086LY9	LSSSHSFPY	11
495	B58ORF_086CW9	CCSHPICCW	11
496	A2ORF_087ML9	MLLPPYLLL	11
497	A2ORF_087LA9	LLPPYLLLA	11
498	A2ORF_087AL9	AQLVSFFPL	11
499	B7ORF_087PL9	PPYLLLAQL	11
500	A2ORF_088AL9	AMAVALSKL	13
501	A2ORF_088CV9	CTTSITLSV	13
502	A2ORF_088LI9	LQAPCTTSI	13
503	A2ORF_088KS9	KLTALFFSS	13
504	B7ORF_088AL9	APCTTSITL	13
505	B7ORF_088VV9	VPSATAMAV	13
506	B7ORF_088SL9	SATAMAVAL	13
507	B7ORF_088VL9	VALSKLTAL	13
508	B58ORF_088LM9	LSVPSATAM	13
509	B58ORF_088LF9	LSKLTALFF	13
510	A2ORF_089SI9	SLSPPSSSI	14
511	B7ORF_089IL9	IPSGLSGPL	14
512	B7ORF_089SS9	SPPSSSIPS	14
513	A2ORF_090VL9	VMLNQFHKL	14
514	A2B58ORF_090FM9	FIYHSQFVM	14
515	B58ORF_090NW9	NSCSFFSCW	14
516	B7ORF_093SL9	SPQQIVLLL	14
517	A2ORF_094FI9	FLLLYIYII	14
518	A2ORF_094MI9	MVQFLLLYI	14
519	B7ORF_094CV9	CPSYLLLQV	14
520	B58ORF_094LF9	LLYIYIIHF	14
521	A2ORF_095VV9	VLQVLLNQV	12
522	A2ORF_097LV9	LIFPIFPIV	15
523	A2ORF_097CI9	CLIFPIFPI	15
524	A2ORF_097LI9	LLLGLVQLI	15
525	A2ORF_097TV9	TIMVLALSV	15
526	A2ORF_097FV9	FLVLLLGLV	15
527	A2ORF_097RL9	RILFLVLLL	15
528	A2ORF_097VL9	VLLLGLVQL	15
529	A2ORF_097KS9	KLTDLITTS	15
530	A2ORF_097AL9	ALSVKLTDL	15
531	B7ORF_097GF9	GPLWIRILF	16
532	B7ORF_097WL9	WIRILFLVL	16
533	B58ORF_097TF9	TSSSARLPF	15
534	B58ORF_097IL9	ITTSSSARL	15
535	A2ORF_098QI9	QLYYYGFSI	16
536	B58ORF_098LY9	LSHISYFSY	16
537	A2ORF_099FL9	FFLYCCWVL	16
538	A2ORF_099FV9	FFFLYCCWV	16
539	B58ORF_099VF9	VLSGYGFFF	16
540	B58ORF_099SY9	SGYGFFFLY	16
541	B58ORF_099GW9	GFFFLYCCW	16
542	A2ORF_100ML9	MVLALSVKL	17
543	B58ORF_101LW9	LYCADICTW	17
544	A2ORF_103VV9	VMTEVLQLV	17
545	A2ORF_103GV9	GQACVMTEV	17
546	A2ORF_103CL9	CVMTEVLQL	17
547	B58ORF_103MF9	MGIGSKDTF	17
548	A2ORF_104FI9	FISPFSIII	18
549	A2ORF_104IV9	IILPLLLLV	18
550	A2ORF_104LI9	LILKEQFFI	18
551	A2ORF_104VV9	VLVSFFKSV	18
552	A2B58ORF_104FL9	FSIIILPLL	18
553	A2ORF_104IL9	ILPLLLLVL	18
554	B58ORF_104VF9	VSFFKSVHF	18
555	B58ORF_104KF9	KLTQSGVNF	18
556	B58ORF_104SF9	SGVNFTHGF	18
557	A2ORF_105YI9	YLCIICFSI	17
558	B58ORF_105HW9	HIYQYYFLW	17
559	B58ORF_105IF9	IMYLCIICF	17
560	B58ORF_105KY9	KIFCHIYQY	17
561	B58ORF_106MF9	MSFFHMLKF	19
562	B58ORF_106HF9	HMLKFSVTF	19
563	A2ORF_110TI9	TMDHTTIAI	18
564	A2ORF_112SI9	SLSPLLPAI	19
565	B7ORF_112TL9	TVLMSSSSL	19
566	B58ORF_112SL9	SSSSLSPLL	19
567	A2B58ORF_114LL9	LTSWMLPGL	19
568	A2ORF_114AL9	AIGTSSFRL	19
569	B7ORF_114KI9	KATLFTIAI	19
570	B7ORF_114TF9	TIAIGTSSF	19
571	B58ORF_114TW9	TSSFRLTSW	19
572	B7ORF_115IL9	IPISFIDML	20
573	B7ORF_116NF9	NPRKMSNSF	20
574	B58ORF_116ML9	MSNSFILKL	20
575	A2ORF_117LI9	LLVPSIVEI	20
576	B7ORF_117LI9	LPCYVLLDI	20
577	B7ORF_119MQ9	MPVSFSCMQ	20

peptide #	***Peptide name***	***Sequence***	**POOL #**
578	B58ORF_120MF9	MSIKPSPSF	20
579	A2ORF_121FL9	FQSGFLLSL	21
580	A2ORF_121SA9	SLLSCITTA	21
581	A2ORF_121LL9	LLGTTFMSL	21
582	A2ORF_121MT9	MIFAFLLGT	21
583	A2ORF_121FS9	FLLGTTFMS	21
584	B7ORF_121AF9	APSPFQRSF	21
585	B7ORF_121GL9	GPFQSGFLL	21
586	B7ORF_121SG9	SPFQRSFAG	21
587	B58ORF_121FF9	FAFLLGTTF	21
588	B58ORF_121FF9	FAGPFQSGF	21
589	B58ORF_121RS9	RSFAGPFQS	21
590	A2ORF_123IA9	IMSTILSPA	22
591	A2ORF_123WA9	WMNTAICTA	22
592	A2ORF_123FT9	FLLKLWMNT	22
593	B7ORF_123SL9	SVAIMSTIL	22
594	B7ORF_123YL9	YPPIPPFLL	22
595	B58ORF_123SY9	STILSPALY	22
596	B58ORF_123AF9	ALYPPIPPF	22
