# Supplementary material for: Influence of HAART on Alternative Reading Frame Immune Responses over the Course of HIV-1 Infection
Source: PLoS One. 2012 Jun 29;7(6):e39311. doi: 10.1371/journal.pone.0039311 (PMC3387156; doi:10.1371/journal.pone.0039311)
Supplement: Table S5 — ARF pooled peptides. Table lists the number of peptide pool, the pool content and the total number of peptides in the pool. (PDF) [file pone.0039311.s005.pdf]

Supplemental Table S5

POOL #	Pool content	number of peptides in the pool
1	TAT and REV splicing variants peptides	2
2	HIV ARF sense A2 and B7 peptides	11
3	HIV ARF sense B58 peptides	11
4	HIV ARF 92 antisense A2 peptides	10
5	HIV ARF 92 antisense A2 and B7 peptides	9
6	HIV ARF 92 antisense B58 peptides	9
7	HIV ARF 128 antisense A2 peptides	9
8	HIV ARF 128 antisense B7 and B58 peptides	8
9	HIV ARF 141 antisense A2, B7 and B58 peptides	10
10	HIV ARF 153 antisense A2, B7 and B58 peptides	5
11	HIV ARF 83, 84, 86, 87 antisense A2, B7 and B58 peptides	10
12	HIV ARF 85, 95 antisense A2 and B58 peptides	9
13	HIV ARF 88 antisense A2, B7 and B58 peptides	10
14	HIV ARF 89, 90 93, 94 antisense A2, B7 and B58 peptides	11
15	HIV ARF 92 antisense A2 and B58 peptides	11
16	HIV ARF 97, 98, 99 antisense A2, B7 and B58 peptides	9
17	HIV ARF 100, 101, 103, 105 antisense A2 and B58 peptides	10
18	HIV ARF 104, 110 antisense A2 and B58 peptides	10
19	HIV ARF 106, 112, 114 antisense A2, B7 and B58 peptides	10
20	HIV ARF 115, 116, 117, 119, 120 antisense A2, B7 and B58 peptides	7
21	HIV ARF 121 antisense A2, B7 and B58 peptides	11
22	HIV ARF 123 antisense A2, B7 and B58 peptides	7
